# Cytomegalovirus Seropositivity and Suicidal Behavior: A Mini-Review

**DOI:** 10.3390/medicina55120782

**Published:** 2019-12-12

**Authors:** Marco Paolini, David Lester, Michael Hawkins, Ameth Hawkins-Villarreal, Denise Erbuto, Andrea Fiorillo, Maurizio Pompili

**Affiliations:** 1 Psychiatry Residency Training Program, Faculty of Medicine and Psychology, Sapienza University of Rome, 00185 Rome, Italy; marcopao88@gmail.com; 2 Psychology Program, Stockton University, Galloway, NJ 08205, USA; David.Lester@stockton.edu; 3 Department of Psychiatry, University of Toronto, Toronto, ON M5S, Canada; mhawkins@shn.ca; 4 Fetal Medicine Research Center, BCNatal-Barcelona Center for Maternal-Fetal and Neonatal Medicine, University of Barcelona, 08028 Barcelona, Spain; amethhawk@gmail.com; 5 Fetal Medicine Service, Obstetrics Department, “Saint Thomas” Hospital, University of Panama, Panama City 0843, Panama; 6 Department of Neurosciences, Mental Health and Sensory Organs, Suicide Prevention Center, Sant’Andrea Hospital, Sapienza University of Rome, 00185 Rome, Italy; denise.erbuto@gmail.com; 7 Department of Psychiatry, University of Campania Luigi Vanvitelli, 80138 Naples, Italy; andrea.fiorillo@unicampania.it

**Keywords:** suicide, CMV, cytomegalovirus, biomarker, antibodies, review

## Abstract

*Background and objectives*: In recent years, a growing body of research has focused on identifying possible biological markers for suicidal behavior, including infective and immunological markers. In this paper, our aim was to review available evidence concerning the association between cytomegalovirus (CMV) infection and suicide. *Materials and Methods*: A systematic search according to the PRISMA statement was performed on Pubmed. After the screening procedure, we identified five relevant papers. *Results*: We found inconsistent evidence linking CMV infection and suicide, with some papers reporting an association between CMV seropositivity and suicidal behavior, and others not finding the association. *Conclusions*: With the evidence available presently, it is not possible to infer whether there is a correlation between suicide and CMV infection.

## 1. Introduction

Although suicide research has made many advances [1], suicide remains an unpredictable [2] but leading cause of death. There are many known indicators/risk factors that can alert physicians’ of those at risk of suicide, including clinical (e.g., psychiatric illness, substance use, previous attempts, medical illness), demographic (e.g., male sex, older age, living alone), genetic, and psychological (e.g., unemployment, interpersonal conflict) factors.

As research advances, many have wondered whether there are biological markers that could help clinicians in the assessment and management of individuals at risk of suicide [3]. In recent years, viral infections have received closer attention as a possible target in suicide research. Among these, cytomegalovirus (CMV) infection has been associated with the development of psychiatric disorders (e.g., schizophrenia and mood disorders) [4,5,6,7,8], neurodevelopmental disorders (e.g., autism) [9,10], neurocognitive disorders (e.g., Alzheimer’s disease) [11], and suicide attempts and completions [12,13,14].

Cytomegalovirus (CMV) prevalence varies by population, with an estimated 40% to 100% of individuals infected [15,16]. Seroprevalence tends to be high in lower socio-economic groups and ethnic minority populations. Because of the high seroprevalence, a large reservoir of CMV continuously exists in the population [17]. CMV remains a major cause of congenital infection and disease during pregnancy around the world. In particular, congenital infection with human cytomegalovirus (HCMV) is a major cause of fetal brain damage [18,19,20]. CMV has a specific neurotropism that is evident from its predominance in central nervous system (CNS) abnormalities observed in symptomatic congenital infection (sensorineural hearing loss, neurological impairments and neurodevelopmental delay [21,22,23,24]).

In children and adults, both primary cytomegalovirus infection and reactivations are typically asymptomatic and, as a result, many people are unaware that they have been infected. The infection can lead, however, to an inflammatory response in the brain both in immune-compromised and immune-competent patients [25,26,27]. Recently, it has been suggested that neuroinflammation and activated microglia play an important role in the pathogenesis of suicide and suicidal behavior [28,29,30].

The aim of the present paper was to review all available evidence regarding CMV seropositivity and suicidal behavior.

## 2. Materials and Methods

**Figure 1:**
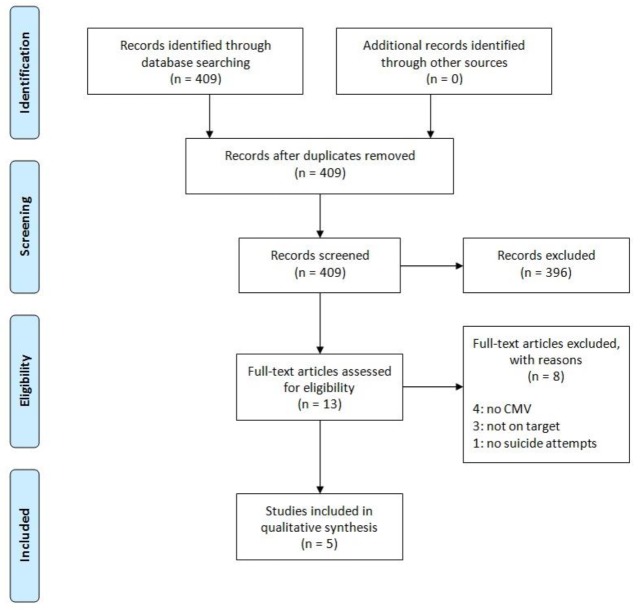
PRISMA flow diagram.

For this review, we followed a systematic procedure in order to identify all peer-reviewed studies concerning CMV seropositivity and suicide. First, we performed a bibliographic search on Pubmed on 14 May 2019, using the following keywords: “(CMV OR cytomegalovirus or herpesvir*) AND (suicid* OR “self-harm” OR “self-killing”)”. We then searched for additional relevant papers after reviewing the works cited in the papers identified, and through the “relevant articles” section on Pubmed. This search resulted in 409 papers. We then applied our inclusion and exclusion criteria. The selection procedure was carried out according to the PRISMA statement (see Figure 1).

In order to be included, papers had to fulfil the following criterion—namely serological studies of anti-CMV antibodies in patients with previous or current suicide attempt(s) versus controls. Our exclusion criteria were: the absence of a control group, the absence of suicidal behaviour in the subjects, and post-mortem studies.

## 3. Results

Of the original 409 papers found, five articles met both the inclusion and exclusion criteria (see Figure 1). Two of these studies were performed on or had a control group drawn from the general population [12,31]; the other three only concerned people with serious mental illness [13,14,32].

### 3.1. Studies in Healthy Subjects

In the first group of papers, an association between CMV seropositivity and attempted suicide was reported (see Table 1). In a case-control study of 12,500 people, Burgdorf et al. [12] studied blood donors from the Danish Blood Donor Study [33], of whom 655 had attempted or completed suicide by the time of the study, 2591 had a psychiatric diagnosis, and 2724 had been in a traffic accidents. These individuals were compared to blood donors without these behaviours or a psychiatric diagnosis matched for age and sex. Burgordorf et al. found that 60.8% of the sample was infected with CMV [12]. The presence of CMV was associated with having any psychiatric diagnosis (OR = 1.17). In the blood donors who exhibited suicidal behaviour (i.e., those with at least one suicide attempt either before or after the blood donation and those who completed suicide after the blood donation), there was a higher prevalence of anti-CMV IgG compared to the controls (OR 1.31, 95% CI 1.10–1.56). However, for people who had attempted or died from suicide only after the blood donation (a nested case control study performed in order to account for temporality and to consider only cases in which the exposure precedes the outcome), CMV infection was no longer associated with these behaviors (OR = 1.18, 95% CI 0.50–2.82). As noted in the paper itself, a possible explanation for this discrepancy could be the small number of individuals having committed or attempted suicide after the blood collection [12].

Zhang et al. (2012) [31], in a cross-sectional observational study on a much smaller sample, compared 54 people who had attempted suicide with 30 controls. No statistically significant association between CMV seropositivity and suicidal behavior was found, although a slightly higher prevalence of anti-CMV IgG was found in people who had attempted suicide compared to the controls (33/53, 62.2% vs. 16/29, 55.1%).

### 3.2. Studies in Psychiatric Patients

Studies performed on people with serious mental illness reported more mixed results (see Table 2). Dickerson et al. [13], in a prospective study, followed 733 patients with schizophrenia spectrum disorders, 483 patients with bipolar disorder and 76 patients with major depressive disorder for an average of 8.15 years. Those who died by suicide (*n* = 16) had significantly higher levels of anti-CMV IgG. Death from suicide was also associated with higher levels of IgG antibodies after adjusting for demographics, psychiatric diagnosis, and psychiatric symptom severity. Suicide risk ranged from 2.51 (95% CI = 0.89–7.10, *p* < 0.082) for individuals with levels greater than or equal to 1 (the cut-off value) to 6.45 (95% CI = 2.15–19.32, *p* = 0.001) for individuals with levels ≥ 3 times the cut-off value. In a multiple regression analysis, male sex, being Caucasian, being separated/divorced and CMV IgG antibody levels predicted suicide.

Dickerson, et al. [14] studied 162 patients with a psychiatric illness including patients with schizophrenia [*n* = 65], bipolar disorder [*n* = 59], and major depression [*n* = 38]. In the total sample, 72 (44%) had a history of attempted suicide. Those who attempted suicide had higher levels of CMV IgM antibodies, and the association was stronger with increasing levels of IgM antibodies (≥75th percentile OR = 3.02, 95% CI 1.08–8.44; ≥90th percentile OR = 6.31, 95% CI 1.17–33.9. *p* = 0.032). No association with IgG class antibodies was reported.

Okusaga et al. [32] studied 950 patients with a diagnosis of schizophrenia, of whom 351 (37%) had a history of attempted suicide. Seropositivity for CMV was not significantly associated with a history of suicide attempt.

## 4. Discussion

The evidence linking CMV seropositivity and suicide is limited. Our bibliographic search resulted in only five papers. Among these, one (Burgdorf et al. [12]) had a much larger sample size than the other four. Furthermore, the identified studies were methodologically heterogeneous, with some comparing CMV seropositivity between those who attempted or completed suicide with healthy controls, and others focused only on people with serious mental illnesses.

When compared to healthy controls, people with a history of suicide attempt(s) seem to have a higher prevalence of anti-CMV IgG, but the effect size seems to be small (Table 1 [12,31]). However, correlation does not imply causality, and, presently, it is not possible to say whether CMV infection is a risk factor for suicide. It may very well be that people with a higher suicide risk are also at a higher risk of contracting CMV infection. In this regard, Okusaga et al. [32] suggested that individuals with suicidal tendencies might engage in activities that increase the risk of exposure to infections—such as not washing vegetables thoroughly or eating undercooked meat.

In regards to people with severe mental illness, the results appear to be mixed. One study reported higher CMV IgG levels in patients with a mental illness (namely schizophrenia, bipolar disorder, and major depression) who died from suicide compared to controls [13], while another study found higher levels of IgM-class antibodies in those who had attempted suicide, but no difference concerning IgG [14]. A third study, carried out only on schizophrenic patients found no association [32].

This heterogeneity of findings could be partially explained by the fact that these studies involved patients with different psychiatric disorders (namely schizophrenia, bipolar disorder, and major depression). People with schizophrenia and psychotic spectrum disorders could have distinct features and a different pathophysiology leading to suicide compared to people with non-psychotic illnesses [34,35]. Other factors that may explain the reported differences between the studies examining the association between CMV infection and suicide is that these studies did not distinguish between acute CMV infection, chronic infection (either congenitally acquired or acquired as an adult), and reactivations of CMV infection. Dickerson et al. [14] suggested that the differences among the studies—in terms of immunoglobulin class—specificity may be related to methodological issues such as the format of the assay and the specificity of the immunoreagents. However, Dickerson et al. did not find that the elevated IgM levels encountered in their study were due to unspecific markers of infection, including naturally occurring antibodies or elevated IgM levels from reactivation from rheumatoid factors.

Previous studies have suggested an association between inflammation and increased suicide risk in people with a psychiatric illness [36], but whether the association between CMV infection and suicide exists is uncertain. Previous research in this area has found increased levels of inflammatory markers (e.g., interleukin-6) in patients with suicidal ideation and behaviour [37,38]. Activated interleukine-6 has been implicated also in acute fetal brain responses and long-term changes in brain development and behavior [39].

Much has been discussed about the effects of CMV infection on the brain of the fetus, but less is known about the effects of this infection on the adult brain, or the degree of brain damage in those with asymptomatic CMV infection. Congenital brain CMV infection is thought to be irreversible. CMV affects many of the brain cells including microglia [40]. Microglial density has been found to be increased in people who have died from suicide. In contrast, no changes in microglial density have been observed between people with a psychiatric illness and healthy controls [41]. Either way, it is too soon to draw conclusions about the potential association between CMV infection and suicide. More studies are needed before causality can be implied.

To our knowledge, this is the first review concerning CMV seropositivity and suicide. The major limitation of this review is that our bibliographic search was conducted using only one database (Pubmed). Another limitation is that our search only resulted in five papers that were methodologically heterogeneous, thus rendering a meta-analytic procedure impossible.

## 5. Conclusions

The evidence linking CMV infection and suicide risk is scarce. To test this association further, studies comparing CMV seropositivity among psychotic and non-psychotic suicide attempters are needed.

## Figures and Tables

**Table 1:** Studies in Healthy Subjects.

Study (Year)	Type of Study	Study Population	Case Population	Control Population	Analysis Method	Outcome
Burgdorf et al. [12]	Case control study	Data from 81,912 individuals from the Danish blood donor study. Total sample: 11,546 cases and controls	Blood donors who died by suicide or engaged in suicide attempts (*n* = 655)	Blood donors who did not die by suicide or engage in suicide attempts (*n* = 6503)	IgG anti cytomegalovirus (CMV). Solid phase ELISA	Seropositivity: 439/655 (67%) vs. 3886/6503 (59.7%), OR 1.31, 95% CI 1.10–1.56
Zhang et al. [31]	Cross-sectional observational study	Suicide attempters from inpatients at Lund University Hospital, Sweden.	Patients admitted for suicide attempt (*n* = 54)	People randomly selected from the municipal population with no psychiatric condition or previous suicide attempt (*n* = 30)	IgG anti CMV, CMV titer. ELISA	Seropositivity: 33/53 (62.2%) vs. 16/29 (55.1%).
		Controls from municipal population of Lund, Sweden. Total sample: 84 cases and controls				CMV IgG titer: 99.5 (±86.9) vs. 91.3 (±92.0)

**Table 2:** Studies in Psychiatric Patient.

Study (Year)	Type of Study	Study Population	Case Population	Control Population	Analysis Method	Outcome
Dickerson et al. 2018 [13]	Prospective study with 16y FU	Individuals with previous diagnosis of schizophrenia spectrum disorder, bipolar disorder or major depressive disorder. Total sample: 1292 individuals	Individuals with serious mental illness who died by suicide (*n* = 16)	Individuals with serious mental illness who did not die by suicide (*n* = 1276)	IgG anti-CMV titer. Solid phase ELISA. Antibody levels expressed as a ratio between the test sample divided by that of a standard control sample.	CMV IgG titer: 3.35 (±3.07) vs. 1.59 (±1.90). Association found between increasing levels of antibodies and Hazard Ratios.
Dickerson et al. 2017 [14]	Cross-sectional study	Individuals with previous diagnosis of schizophrenia spectrum disorder, bipolar disorder or major depressive disorder. Total sample: 162 patients	Individuals with serious mental illness with previous suicide attempts (*n* = 72)	Individuals with serious mental illness without a previous suicide attempt (*n* = 90)	Anti-CMV IgG and IgM titer. Solid phase ELISA.	Association between suicide attempts and IgM anti CMV measured as a continuous variable (coefficient 0.151). Increased odds of suicide for levels of IgM anti CMV ≥ 75th and 90th percentiles (OR 3.02 and 6.31 respectively). No association with IgG.
Okusaga et al. 2011 [32]	Cross-sectional study	Patients diagnosed with schizophrenia through SCID, recruited in the Munich area of Germany. Total sample: 950 patients	351 individuals with schizophrenia with previous suicide attempts.	599 individuals with schizophrenia with no previous suicide attempt.	IgG anti-CMV. Solid phase ELISA.	Seropositivity for CMV not associated with a history of suicide attempt. No further data provided.
